# Supplementary material for: Holistic acceptability of an adult levofloxacin formulation in children and adolescents on a tuberculosis preventive treatment trial
Source: PLOS Glob Public Health. 2024 Jul 5;4(7):e0003381. doi: 10.1371/journal.pgph.0003381 (PMC11226063; doi:10.1371/journal.pgph.0003381)
Supplement: S1 Text — (DOCX) [file pgph.0003381.s001.docx]

| Title of the Research Project: Acceptability of adult formulation levofloxacin for children and caregivers on an MDR prevention therapy trial  A single-site, in-depth, qualitative study nested in TB-CHAMP and exploring a new conceptual framework of antituberculosis treatment acceptability  Researchers Name(s): Dr Susan Purchase  Address: Desmond Tutu TB Centre, K-Floor, Clinical Building, Tygerberg Campus, Stellenbosch University, Parow  Contact Number: 021 938 9686  **Child-Caregiver Discussion Guide** |
| --- |

**Purpose:** To assess the acceptability of the adult formulation of levofloxacin/levofloxacin placebo (study medicine) to caregivers and children on the TB-CHAMP trial

1. To explore the children and caregivers’ experience of the study medicine, including its palatability, administration processes required, and its appeal.
2. To explore the children and caregivers’ perceptions of TB and TB preventive therapy (TPT), including conceptions of health and illness and responses to adverse effects.
3. To explore the child’s experiences relative to their family’s history of experiences with TB.
4. To explore treatment processes involved in TPT care, including any barriers or facilitators experienced during health systems access.
5. To explore the psychosocial impact of the study drug regimen on child/caregiver, including financial implications of care.

**Form of data recording:** (1) Audio-recording of all talk from “Preamble” to “Closing”; (2) Reflexive tools to be completed in the form of a reflective document (post interview) and an activity report template (during interview)

**Expected time needed per use:** Approximately 30-60 minutes

**Instructions for use:** Questions and probes should be used flexibly with each participant. Questions are phrased to be posed to caregivers, but should be adapted when an older child is interviewed

**Preamble** (to be read by facilitator at the start of each encounter): Today is the (insert date [day xx^th^ Xxx xxxx]) and it is (insert time XX:XX) and we are in (insert site). This is a discussion with a child/caregiver enrolled in the TB-CHAMP trial. Thank you for your time. May I remind you that we are audio recording this discussion and ask that you speak loudly and clearly? I would also like to remind you that your taking part in this research is entirely voluntary and you can withdraw from this study at any point. As the facilitator I will also be taking some notes. Do you have any questions before we begin?

**Visit 1**

# Understanding household’s experiences with TB

# Activity 1: Kinship map activity

**Aim:**

To draw a picture that represents (1) the participants’ “family” (biological and non-biological), (2) the types of relationships between each member of that “family”, and (3) cohabitation/co-residence of people (“family” and non-family) with the participant.

**Key:**


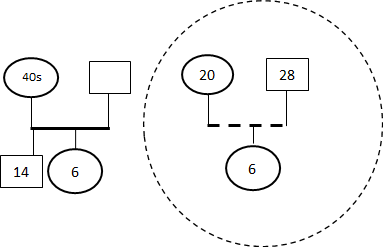


|  | Woman/Female |
| --- | --- |
|  | Man/Male |
|  | Formal Relationship |
|  | Informal Relationship |
|  | Formal Relationship Ended |
|  | Informal Relationship Ended |
|  | Relation/Relationship |
|  | Deceased |
|  | Co-residence |

**Steps for Researcher:**

1. Make sure you have a pencil, eraser, plain paper, sharpener, and clipboard.
2. Begin by getting the participant to be as comfortable as possible. Ways in which this can be achieved is to assure the participant that they are doing this because their story may help us to better develop treatment and care for others with the same health condition. To do that, we need to really understand people’s lives and where illness fits in. Have them tell you about a friend and explain that if it makes them feel better, they can imagine talking to that friend.
3. Researcher to tell the participant: “We are going to be doing a Kinship Map with you today. A kinship map is like a family tree, or something we use to draw your family and household. It will help us to see who you live with and help us to get to know you and your family. We are going to draw the family tree or kinship map together.”
4. Ask the participant to create a list of people that are part of their family (this must include at least three, but ideally four generations). Remember to note ages (or estimated ages as well).
5. Next you will help the participant to list the members of their household.
6. Draw a circle or square to represent the participant, following the key provided above. Indicate names and ages.
7. Ask about their parents and draw the parents, including the relationships between the parents.
8. Ask about any siblings and draw each sibling and the relationship between each sibling and the participant’s parents or the siblings’ parents (if not direct biological siblings).
9. If not the direct biological sibling, use the key to demonstrate the type of relationship between the sibling and parent in relation to the participant.
10. Ask the participant if they have any children and, if relevant, using the key, indicate the relationships to their offspring’s co-parent.
11. Ask about family dynamics such as the relationship the participant has with each family member.
12. Check that you have the participant’s entire family represented on the kinship map. This should include at least three, but ideally four generations (the participant and siblings/cousins, parents, grandparents, and children/nephews/nieces of the siblings) AND a clear indication of the types of relationships between each member of this kinship map (using the different types of lines as per the key above).
13. Include names and ages
14. Ask about co-residence/co-habitation. i.e., who sleeps in the same household/dwelling as the participant on a regular basis. Draw a dotted circle to include these co-residents. Ensure that the participant has not forgotten anyone who is co-resident, even if not related.
15. Tell the participant how you think you understand their “family”. Show the participant what you have discussed and allow them to correct you if any details are wrong.
16. Once all is correct take a picture of the kinship map.
17. After the discussion is concluded, redraw the kinship map in the office in PowerPoint, submit and save to the data PC. Remember to add the ages of each participant on the kinship map.

## **Study specific probes:**

## Could you please tell us about your household:

### How many people live in your home? How are they related to you/your child?

### Are all household members and family members aware that the child is on study drug and that the child is enrolled in TB-CHAMP?

### How has your child’s inclusion in the study, impacted the relationships within the home? (Are there people who have since moved out? Are there people whose roles have changed?)

### Have new members joined the household since the child started on TB-CHAMP? (Are there people assisting you as the caregiver? Are you the only caregiver to the child?)

### Has anyone in your family had TB before? Can you tell me more about that? (When did they get TB? What kind of TB? How long were they on treatment?)

# Activity 2: History of TB in family timeline activity

**Aim:**

To understand your family’s history of TB and other diseases.

**Steps for researcher:**

1. Researcher to participant: “We are going to be doing an illness timeline activity. An illness timeline allows researchers and healthcare workers to understand what illnesses you and your family have experience of living with – in this case ,TB. This also allows researchers and health workers to understand the role TB plays in your family’s life and the impact TB has had from one generation to the next. The timeline will allow us to see the ‘main/important events’ that has occurred in your family. “
2. Show the participant the example of the timeline (Appendix X) and explain that a timeline is a way of showing/telling a story with remarkable events in time marked.
3. Using the Kinship Map (description) ask the participant if anyone in their family is currently living with a disease. Plot the points such as diagnosis, symptoms, referrals, and major events next to that person (even if deceased).
4. Ask the participant to tell you the story of the next family member living with a disease.
5. Ask the participant to talk you through any family member’s specific experiences of TB. Ask the participant to include details as above: diagnosis, symptoms, referrals, and major events next to that person (even if deceased). Assist where needed.
6. Continue asking the participant to indicate each ‘beat’ along their family members’ experiences of living with TB and note these along the timeline. For now, only capture a ‘heading’/short description of what the event was and when it happened. Later you will come back and talk about each moment in more detail. For now, just try and capture the overall story.
7. Once you have to overall story (point-by-point), narrate it back to the participant so that they can add events they may have forgotten or clarify your understanding.
8. Refer to each of the family members and highlights along the journey, probing for more details of each family member (in turn). Ask for details on the story (who was there, what happened, how did they feel, what were they worried about).
9. Refer to the questions and probes below if the interview requires a few questions to assist the participant if they get ‘stuck’.
10. Once you have spoken about each family member in the Kinship Map, ask the participant to look over the timeline.
11. Once the participant is satisfied with the information captured on the page and the researcher does not have any follow up questions on the participants experiences.

**Probes (on timeline):**

Tell us what happened at this point

Tell us what your reaction to this was

Tell us about how you felt at this point

Tell us who was involved and how

Tell us how it affected life after it happened

Tell us about your family life after this (was there a long adjustment period? How does your family treat you now? etc)

## **Study specific probes:**

## Could you please tell us more about your child’s treatment journey?

### When did you first learn about TPT?

### What did they tell you about TPT at the clinic?

### Did your child receive TPT from a local clinic before they started on TB-CHAMP? Tell me about that.

### If so, how long was your child on that treatment? Tell me more about the treatment. (Was it easy to prepare and administer? Did your child like the treatment? What did it taste like? What are some of the challenges you experienced with the TPT you received from the clinic?)

# Activity 3: Social network activity

**Aim:**

To create a platform to discuss and understand the participants’ social network and the relationships the participant engages in, by visually representing kinship, friendship, romantic, communal and acquaintance networks of the participant.

**Steps for the researcher:**

1. Ensure that you have pens, paper, clipboard, and highlighters ready.
2. Researcher to tell participant: “Our next activity is a social network. A social network map is something we use to draw/capture your significant relationships or the people that you have different types of social relationships with. It will help us to see who are the people that are closest to you and your friendship dynamics.”
3. Ask the participant to either pick an avatar/draw an image/symbol to represent themselves or write their name at the centre of a page (see example on Appendix X below).
4. On separate pages, ask the participant to write down a list of the groups that they form part of/places that they frequent i.e. school, work, religious establishments, youth centre, sports clubs or any other group/place that the participant lists (this is not limited to physical groups, it may include mobile groups like WhatsApp or Facebook or other social network platforms that they form part of).
5. On each page under each heading the participant will then write down the names all the people that they engage with in these groups/places. Continue prompting until the participant has listed all the people with whom they socialise with in each respective circle. DO NOT YET ask them any probing/follow-up questions about their relationship or experiences with the person/people.
6. Where the participant chose their avatar/ image /symbol you will draw the first group in a bubble as an example to the participant (See Appendix X example below).
7. Then give the marker to the participant and ask them to add all the other groups (bubbles) that they have social connections.
8. In each bubble the participant will then write down each of the names that have previously listed as part of that group (refer to the example attached).
9. Confirm with the participant if the drawing is an accurate representation of their social network – amend, as necessary.
10. Explore the links between groups by using a highlighter to indicate overlaps. If there are people that are present in multiple bubbles use a highlighter and highlight the person’s name that overlaps in multiple bubbles.
11. Now return to each person to whom they have listed and, in turn, ask them to describe how they came to know each one of the people that they have listed and their relationship to the person.
12. Take a picture of the network.
13. Ask follow-up questions and probes specific to the study.
14. After the discussion is concluded, redraw the social network map in the office in PowerPoint, submit and save to the data PC.

## **Study specific probes:**

## Could you please tell us more about your social network (friends and community):

### What is your community like? (Do people spend a lot of time together?)

### How often do your friends come over? Where does your child spend a lot of time? Who does your child spend a lot of time with?

### Did anything change with your friends, when [index case] found out they had MDR-TB?

### Did anything change with your friends, or your child’s friends, when your child started taking the study drug?

# Activity 4: Weekly timeline activity

**Aim:**

To identify the ways in which participants structure their daily lives by (1) exploring the regular day-to-day activities participants engage in; (2) to describe the places participants visits during the week or weekend; and (3) to describe who they hang out with during these activities.

**Steps for the researcher**

1. Researcher to participant: “We will be doing a timeline of what you do and where you go during a ‘regular’ week. A weekly timeline allows researchers to understand what your daily routine looks like and to understand if and how living with [health condition] might have affected your lifestyle.”
2. Using the template (Appendix x), with Sunday at the left-hand side (start) of the line, and Saturday at the right-hand side, the researcher should explain to the participant that they should list all of the things that participants usually do on all of the given days, under the written name of the day. Researchers should provide an example to ensure the participant understands what is expected from them in this activity.
3. Ask the participant to list everything that they do on Sunday, until Saturday while capturing it on a timeline. Probe on the places they visit and they people they do these activities with.
4. Once you have spoken about each event on the timeline in detail, ask the participant to review the timeline, and ask if there is anything that is missed.
5. Take a photo of the timeline.

# Visit 2

# Activity 1: Treatment adherence timeline activity (for index case – if caregiver/household member)

**Aim:**

To understand the challenges/motivations that may contribute towards treatment adherence for the participant

**Steps for researcher**

1. Researcher to participant: “We will be doing a treatment journey timeline activity to capture the ups and downs of taking [illness/condition] treatment. We want to understand some of the challenges that you experience in taking our treatment on a regular basis and the reasons why people sometimes stop taking their treatment.“
2. First mark the end point which is the date when the activity is conducted, as well as the starting point which is when the participant initiated their treatment first time – use Appendix X, Standard timeline activity template, page 12.
3. Ask the participant about the treatment interruptions they have had throughout their treatment journey and to indicate them on the timeline.
4. Thereafter, ask the participant to indicate the events that had helped them get back to adhering to treatment each time they stopped or interrupted taking treatment.
5. At this point, you just need to mark each of these events on the timeline only to note that they exist and DO NOT dwell on details yet.
6. Make sure the treatment journey events include remarkable times when they missed doses or stopped treatment for a longer period, when they restarted treatment, when they were most consistent or inconsistent on taking treatment and so on.
7. Once the participant marked these treatment journey events on the timeline, discuss more details about each event in turn with the participant and make notes on the timeline.
8. Once you have written and spoken about each event on the timeline in detail, summarise their overall treatment journey and ask the participant to look over the timeline to confirm details.

**Probes (if necessary):**

- Tell us what happened at this point
- Tell us what your reaction to this was
- Tell us about how you felt at this point
- Tell us who was involved and how
- Tell us how it affected life after it happened
- Tell us about your family life after this (was there a long adjustment period? How does your family treat you now? etc)

# Activity 2: Treatment adherence timeline activity (for TPT patient)

**Aim:**

To understand the challenges/motivations that may contribute towards treatment adherence for the participant

**Steps for researcher**

1. Researcher to participant: “We will be doing a treatment journey timeline activity to capture the ups and downs of taking [illness/condition] treatment. We want to understand some of the challenges that you experience in taking our treatment on a regular basis and the reasons why people sometimes stop taking their treatment.“
2. First mark the end point which is the date when the activity is conducted, as well as the starting point which is when the participant initiated their treatment first time – use Appendix X, Standard timeline activity template, page 12.
3. Ask the participant about the treatment interruptions they have had throughout their treatment journey and to indicate them on the timeline.
4. Thereafter, ask the participant to indicate the events that had helped them get back to adhering to treatment each time they stopped or interrupted taking treatment.
5. At this point, you just need to mark each of these events on the timeline only to note that they exist and DO NOT dwell on details yet.
6. Make sure the treatment journey events include remarkable times when they missed doses or stopped treatment for a longer period, when they restarted treatment, when they were most consistent or inconsistent on taking treatment and so on.
7. Once the participant marked these treatment journey events on the timeline, discuss more details about each event in turn with the participant and make notes on the timeline.
8. Once you have written and spoken about each event on the timeline in detail, summarise their overall treatment journey and ask the participant to look over the timeline to confirm details.

**Study specific probes (if necessary):**

- Tell us what happened at this point
- Tell us what your reaction to this was
- Tell us about how you felt at this point
- Tell us who was involved and how
- Tell us how it affected life after it happened
- Can you tell us about your child’s experience of taking the study medicine?
- Have there been times when the study medicine has been easier and more difficult to take/administer? Why? What changed?

# Activity 3: Administering medicine to a doll activity

**Aim:**

The aim of this activity it to allow younger participant (children) to show you how medication is administered to them. This will also provide an opportunity for the researcher to ask questions about how they feel when they take their medication and the taste thereof.

**Steps for the researcher:**

1. Make sure all the medical kit toys / action figures / dolls are sanitised and clean.
2. Place them on the table in front of the participant.
3. Explain to the participant what the activity entails and allow the participant to select a doll or action figure of their choosing.
4. Ask the participant if they understand what all the ‘tools’ are in the medical kit. If needed, tell them which one is the tablet, capsule, medicine bottle and syringe.
5. Tell the participant to show you how they take their medication by using the toys and doll to show you.
6. Illustrate to the participant using a doll and capsule, that some children who have [health condition] may not be able to take the capsules themselves and a nurse/parent/caregiver uses different ways to give the medication. Show the participant that it can be crushed, diluted or injected (depending on the medication). Now they should try showing you how they take their medication.
7. If the participant struggles you may ask questions such as ‘which of these toys looks like the medication you take?’ and allow them to choose and say it out loud.
8. Once the participant has chosen an object, tell them to show you how they must take it (there may be various forms and actions involved) repeat their action back to them, verbally, for confirmation.
9. Ask the participant how they feel when they must take their medication and whether it hurts (during or after taking the medication). They may also show you where it hurts by using the doll / action figure to point to these places.
10. Ask the participant what the medication tastes like when they drink it.
    (Depending on the age of the participant you may need to probe or ask their caregivers).
11. Once the activity is done, use the study specific questions and probes for further information. Once all questions and probes have been answered, conclude the activity.

# Study-specific probes re: usability, palatability, and adherence to study drug

## Could you please tell us more about the study medicine your child is taking?

### Have you or your child ever taken medicine to prevent or treat TB before? (If not, do they know anyone else who has been on TB preventive therapy or TB treatment—where they’ve actually seen the treatment?) Can you tell me about it? How many pills did you/your child have to take? For how long? What were some things you disliked about taking TB medicine?

### How does your child feel about taking the study medicine? Do you have to convince your child to take the medicine? (For example, do you have to bribe them?) Or use force? Or distract them? Or reward them?

### When during the day does your child take the tablet?

### Do you think it would be better to swallow the pill whole or is better to dissolve it in water? How much water do you use to dissolve it in? Do you think there are other ways to take the medication?

### Questions to older children:

### What does the study drug taste like? Does it matter if you have already eaten something before/after taking the tablet? What are some of the worst tasting things that you can’t stand to eat/drink, and how does this tablet compare?

### If you were going to tell another child about this tablet, how would you describe it? What ideas do you have to explain what it tastes like?

# Activity 4: Instructions for the Ranking activity – Barriers

Aim: To identify what participants think are the barriers to (implementation of health services, treatment, adherence, disease etc.) by: (1) representing a list of barriers (2) organise the list of barriers from the most difficult to the least difficult.

**Steps for the researcher**

1. Set out a piece of paper on the floor or table
2. Provide the participant with post-its
3. Ask the participant to think about the challenges or barriers that they experience accessing health services by saying: I would like us to write down all the challenges/barriers that you can think of on the post-its.
4. Once the challenges/barriers have been written down on multiple post-its prompt the participant into ranking the barriers, starting with the one that they think poses the biggest challenge to health services access to the barrier that they find the most manageable.
5. Once the participant has ranked the challenges/barriers ask the participant to provide reasons for their rankings and to provide examples where possible. Ask the participant “Why did you place X barrier above or below Y barrier. Why is X barrier a bigger problem than Y challenge/barrier?”
6. Once, the participant is satisfied with their rankings take a picture of the barriers, rankings.
7. Now use the questions/probes in the discussion guide to explore their experiences further.

Come back to the office and take another picture of the card sorting activity (take care to make these as clearly as possible, submit and save to the data PC).

# Activity 5: Body map activity

**Aim:**

To draw a picture that represents (1) the participants’ “body”; (2) to explore how the participant perceives their body; 3) to understand how the participants experience their health condition(s); and 4) to explore the participant’s emotional-physical-social experience as a result of their health condition(s), including the treatment they receive.

**Steps for researcher:**

1. Researcher to ensure that they have a large piece of paper (+/- 2m) and coloured markers/pens and their camera available.
2. Researcher to participant: “We would like to understand how [health condition] has affected your body. To do that, we are going to draw a ‘body map’. A body map is a life-sized outline of your body where you can fill in or draw details.”
3. Ask the participant to lie down on a large piece of paper so you can draw the outline of their body. If they are unwilling/unable, draw the outline of one of the researchers or draw freehand.
4. Ask the participant to mark all the places on their body where their condition affects them. This can be done using words, symbols, shapes, or pictures.
5. Ask the participant to elaborate on why they have chosen that type of symbol for each of the ‘symptoms’/impacts.
6. Ask the participant to expand on the symptoms of their condition as indicated on the body map.
7. Ask the participant to expand on how the treatment that they are taking makes their body feel.
8. Tell the participant how you think you understand their “body”. Allow them to correct you if any details are wrong.
9. Once all is correct take a picture of the body map.
10. Now use the questions/probes in the discussion guide to explore their experiences further.
11. After discussion has been concluded, take another picture of the body map. Ensure that the picture is as clearly as possible, submit and save to the data PC.

# Study-specific probes re: adverse effects of study drug

## Could you please tell us more about how your body has changed/been affected since you/your child started using the study drug?

### What are some of the difficulties and challenges you and/or the child experiences taking study medicine?

### Are there any problems with the medicine (side effects)? If yes, what are they and what do you do? Do you have any questions regarding these side-effects?

# Visit 3

**Note: Ideally a home visit**

# Activity 1: Disclosure map activity

**Aim:**

*To create a platform to discuss the participants’ disclosure practices by knowing to whom they have disclosed their illness/condition, who else knows about their health condition, and who they do not want to know.*

**Instructions for researcher:**

1. Researcher to participant: “We will be completing a disclosure network activity which is a representation of who in your life knows about you/your child being on a TB prevention study and taking the study drug. We want to understand why and who you chose to tell about being on the study. This will also allow us to understand how people make decisions around sharing their health status.”
2. Using Appendix x below, ask the participant to draw an image/symbol to represent themselves or write their name at the centre of a page.
3. Around this, draw four concentric circles (refer to or use the template)
4. Ask the participant to list all the people who had not known that they (the participant) were on a TB prevention study and to whom they (the participant) disclosed about the study. For the first person they mention, confirm that this was actually an active disclosure, not someone who just knew already (e.g., their nurse). For each person that the participant lists, get them to write their name/symbol in the circle closest to the centre of the page/the participant’s symbol/name. Continue prompting until the participant has listed all the people to whom they have actively disclosed. DO NOT YET ask any probing/follow-up questions about their disclosure experience with this person.
5. Ask the participant to list all the people to whom there was ‘passive’ disclosure, i.e. people who learned that the participant was on the study without the participant having to tell them. This includes people to whom there was accidental disclosure or who found out without the participant wanting them to. For each person that the participant lists, get them to write their name/symbol in the second circle. DO NOT YET ask them any probing/follow-up questions about their disclosure experience with this person.
6. Ask the participant to list all people who they (the participant) believe ‘strongly suspect/know’ that the participant is on the TB prevention study, but that this belief has not be confirmed. For each person that the participant lists, get them to write their name/symbol in the third circle. DO NOT YET ask them any probing/follow-up questions about their disclosure experience with this person.
7. Lastly, list all the people who the participant hopes do not know that they are on a TB prevention study. Write these names in the outer circles (furthest away from the participant’s name/symbol).
8. Confirm with the participant if this network is an accurate representation of who knows, how they know, and who does not know – amend, as necessary.
9. Take a picture of the network.
10. Now go back through each person to whom they have disclosed (active or passive), in turn, and ask them to tell the story of the disclosure experience (where, when, how, what was their reaction, what words did they use, how did the participant feel, why did they decide to disclose).
11. For the people who they suspect know, ask them to explain why they suspect that these people might be aware of their diagnosis.
12. For those who do not know their diagnosis, ask the participant why they do not WANT these people to know about their status.

Take a picture of the disclosure map.

## Study specific probes re: Impact of TPT/study medicine on household

### Can you tell me about how your /your child’s study medicine/participation in study has affected your family, household, and relationships?

#### Have you told people outside your household that your child is on the study? Why/why not?

#### Are there people who you do not want to find out that your child is on the study? Why do you not want these people to find out? What will happen if these people found out?

*[Note: These 2 questions probably already covered]*

#### How did people react when you told them that your child was on the study after being exposed to MDR-TB?

#### Has anyone said anything or done anything to you or your child, that you didn’t like since your child started on this study?

**Appendix: Disclosure network**

Participant

name

# Activity 2: Household floorplan activity

**Aim:**

The aim of this activity is 1) to draw the infrastructure of the participant’s house and spaces that they use on a daily basis; 2) to understand the participant’s perception of their home and how they relate to their house/home; 3) to understand the participant’s perception of how other people experience their house/living space.

**Steps for researcher:**

1. Make sure you have a pencil, eraser, plain paper, sharpener, and clipboard.
2. Researcher to participant: “As part of this activity we will be drawing a floorplan for your house to better understand the layout of your home and how different people use the space. This will help us understand if the ways in which household structures influences the ways in which they manage their health or illnesses.”
3. Ask the participant to draw an outline of their house and the yard overall. Show them an example (see below).
4. Help the participant to draw the inner lines of the house, lines that separate rooms and passages as well as windows and doors. Show them the example.
5. For each space, ask the participant to draw the furniture (example, if they have these items, TV, beds, stove, etc).
6. Discuss the floorplan with the participant. Ask them to correct you where you may have misunderstood.
7. Add the (estimated) dimensions of the room in metres.
8. Ask the participant to explain how different spaces are used/shared (sleeping arrangements, cooking, etc.). Ask if these sleeping arrangements change during different times of the week/month/year (e.g. if different people join/leave the household).
9. Ask the participant who makes decisions around who sleeps where.
10. After the discussion, follow up with your prepared probes (if relevant).
11. Discuss the floorplan with the participant, again. Once they have corrected your misunderstandings/ parts you missed.
12. Take a picture of the plan.
13. After completing the discussion, return to the office and draw the floorplan in PowerPoint using the template. Ensure that all details are captured. If there are elements in the house not indicated with the key, add a blank blocks/quare and add text with details. “Group” all elements of the floorplan and save as “Image” as well. Submit and save to the data PC.

**Study specific probes re: household structure and changes**

## The next questions are to understand how being on the TB prevention study has impacted the relationships, finances, or space in your household?

### How has the child’s inclusion in the study impacted how relationships, finances, or space are being used in the household?

*[Note: Please probe around study financial compensation, and impact this has had]*

### Have there been any unforeseen or additional changes while your child has been on the study?

### (If the index case is part of the household) Has [index cases’] treatment and care needs impacted the household? If so, how?

### Has [index case] been more of a burden than before they were diagnosed with MDR TB?

### Have you had to change the way you prioritise (or spend) your time, money or other resources, since [index case] started treatment?

**Key:**

**
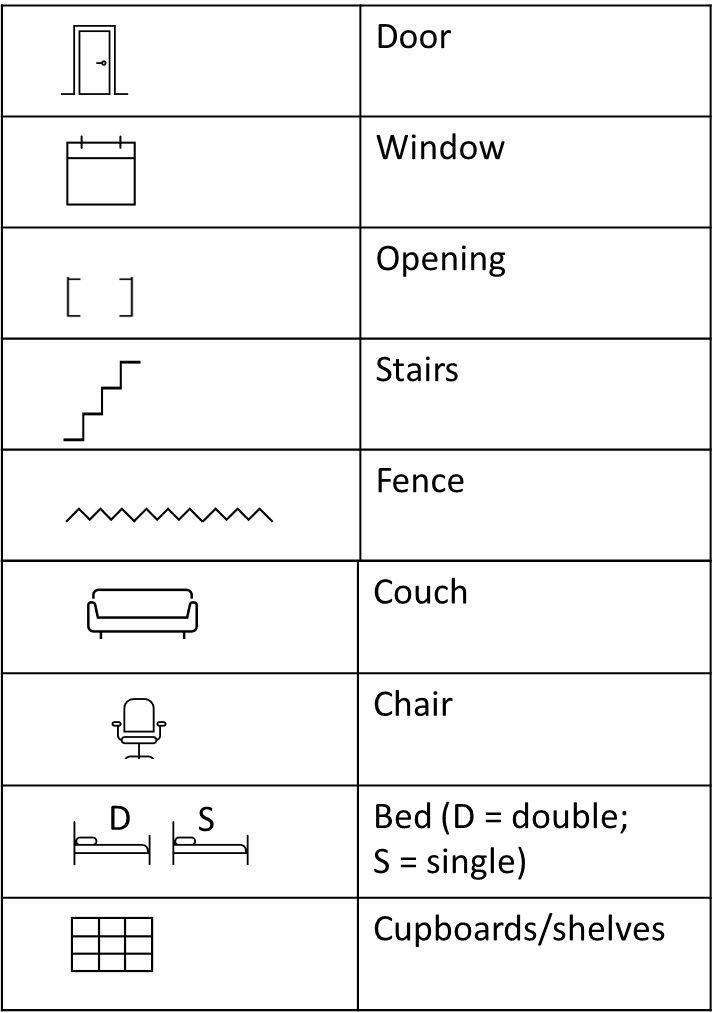

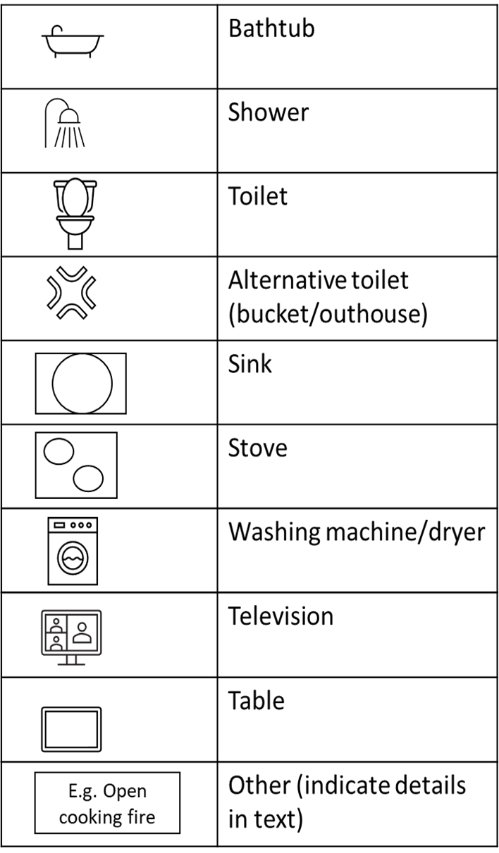
**

*[Note: Floorplan will need to be drawn in PP]*

# Activity 3: Health beliefs: Health belief systems

**Aim:**

The aim of this activity is to understand how participant makes sense of and manage their health. By discussing the different health systems that participants engage with, we are better able to understand how participants make decisions around accessing care or treatment. To do this we will ask participants to (1) identify the different health systems they access and (2) explore how participants engage with these health systems for varying health conditions.

**Steps for the researcher:**

**Part 1**

1. Provide the participant with an explanation as to the purpose of the activity by saying: “We want to understand the different types of services that people use when they are feeling ill or when they are concerned about their health or well-being.” Explain to the participant that people have different preferences for managing different health conditions. “These preferences are based on the things that people believe, their previous experiences, as well as advice and beliefs from the people around them.”
2. Using the template below (Appendix x) ask the participant to look at the bubble with the word “STAYING HEALTHY” and then ask the participant to tell you about the things they do, to stay/ maintain good health (exercise, eating certain foods, drinking water, vitamins, herbs, habits, etc.) List these ways under the bubble.
3. Ask the participant to look at the next bubble with the heading “HEALTH BARRIERS” Ask the participant to tell you about the things that hinder their ability to maintain good health (Probe on Demographic and Socioeconomic factors) list these barriers under the bubble.
4. Ask the participant to look at the next bubble with the heading “ILL HEALTH ACTIONS”. Have the participant tell you about the things they do when they feel sick. (Who do they tell? Where do they go? Are there things they avoid doing when they are not well?) List the things they do under the bubble.

**Study specific probes re: beliefs/understanding of TB**

## The next questions are to understand more about what you think about TB and TB treatment.

### When I say Tuberculosis (TB), what is the first thing that comes to mind? When did you first learn about TB?

### How do you think people get TB? Is there any group of people who are more likely to get TB than others? Why?

### How do you understand MDR TB? How is it different to drug sensitive TB?

### What are some things a person can do to prevent themselves from getting TB?

### What was your reaction when you heard that your child could start on this study?

### What do you think the differences are in the treatment your child is receiving compared to the medications used to treat TB disease?

**Appendix:**

# Activity 4: Health beliefs: Health beliefs Action activity

[Note: you need to print rating scales]

**Aim:**

The aim of this activity is 1) to understand the thought process that influences health seeking behaviours in participants; 2) to understand how perceptions of susceptibility and severity of disease influence choices to prevent disease and adhere to treatment; and 3) to understand how individual’s perceptions of how they SHOULD behave, influences their eventual decisions to either prevent a disease and/or adhering to treatment.

**Steps for researcher:**

1. Show the participant the Rating Scales (see below).
2. Explain that the scale represents two extreme answers, and, depending on the question, they will need to indicate with a marker where they would locate their answer in relation to the two extremes (more towards the one end or the other end). Then give the participant the page, a marker.
3. Note what type of behaviour (see 4) is relevant to this participant on the rating scale page where indicated.
4. Start with the first rating scale (susceptibility) and ask the participant to make their mark on the scale.
5. After the participant has made their mark on the scale, first probe:
   1. **Why they made the mark there.**
   2. **Ask the participant to give examples from their life to support this choice (where they put the mark)**
   3. **Ask the participant to explain how they feel about this and if there is anything that they think they can do to change this.**
6. Then move on to the next scale (severity, locus of control (general), control over the specific behaviour, perceived descriptive norms, and perceived injunctive norms) and repeat the robes listed above. Conclude one set of questions before moving onto the next set.
7. Once you are done with scales discuss with the participant and be certain you are both satisfied with the information captured.
8. Specifically ask if this reflects how they make choices about [prevention] or [adherence/treatment]

**Rating scales – Prevention**

For each statement, indicate how strongly you agree/disagree:

1. Susceptibility

If I use TB preventive therapy, then I will probably **NOT** get MDR-TB disease.

**Strongly disagree_______________________________________________________Strongly agree**

1. Severity

If I were to get MDR-TB then that would be bad.

**Strongly disagree_______________________________________________________Strongly agree**

1. Control over outcomes (general)

In general, in my life, what happens to me is a consequence of the choices I make, not fate.

**Strongly disagree_______________________________________________________Strongly agree**

1. Ability to implement behaviour (specific)
   1. I know what things I need to do to prevent MDR-TB.

**Strongly disagree_______________________________________________________Strongly agree**

- 1. It is easy for me to prevent myself/my child from getting MDR-TB

**Strongly disagree_______________________________________________________Strongly agree**

# Activity 5: Parameters of ambitions activity

**AIM:**

To understand the parameters of ambition of participants which take the form of hopes, dreams, ambitions, and fears. By discussing these parameters, we may illicit the motivations for general health seeking behaviour such as pursuing/not pursuing health care, adherence, and treatment acceptability. The participants response to this activity aids in understanding their experiences of illness, adherence, disclosure, and other health related experiences and assists in the progress of treatment programmes.

**Steps for researcher:**

1. Ask the participant to imagine their ideal self/the self they wish they would be/could be (fantasy they have about themselves). Ask for details for the story of their ideal achievements, characteristics, who they would associate with or be friends with, where they would live, etc.
2. Provide the participant with the ‘bubbles’ picture below and a pen and ask them to fill in details on the ‘ideal self’ on the page
3. Ask the participant to tell you what /how big the difference between the ideal and actual self is.
4. Ask the participant about major achievements/dreams achieved up to this point in their lives.

Timeline activity:

1. Tell the participant: “We have done several timeline activities about things that have happened in the past. Now we would like to do a timeline about the future”. Ask the participant to draw a timeline to indicate their plans and dreams for the future.
2. Ask the participant what they want to achieve within the next year and long-term (within the next five to ten years). Indicate on the timeline.
3. Ask the participant if they have taken any steps to achieve these goals, or if anyone/anything has helped them towards these goals.
4. Ask them to tell you what challenges/fears that hold them back from not pursuing these dreams/ambitions (i.e. illness challenges, financial strains).
5. After, ask them what motivates them to hold on to their hopes /dreams despite the challenges.
6. Ask if the participant had any dreams/hopes when they were younger that did not work out as expected or that they think will not be achieved. Probe for details.

## **Study specific probes:**

## How do you think (if at all) the study and TB in your household has affected your/your child’s future?

### Did the study/study medicine affect your/your child’s schooling?

### Did the study/study medicine affect your/your child’s ability to participate in sports, or other things they usually enjoy doing?

### What are there some of the things you are worried about with regards to your child’s future?

## Can you tell us about any current concerns you have about your/your child’s general wellbeing?

### How do you think the study medicine you/your child received in the trial affected their health?

### 1.10.2 **To caregiver**: Did your child ever seem down/depressed? Were you concerned about their mental wellbeing?

### **To adolescent**: Did you ever feel down/depressed? Did you/your caregiver feel concerned about your mental wellbeing?

**Appendix:**

**Appendix:**

# Activity 6: Placebo

This activity aims to gauge caregiver/adolescents’ understanding of a placebo, and their perceptions of being involved in a placebo-controlled trial. Tell the participant you are going to end the interview by asking them some questions and then having some fun!

Study specific questions/probes:

1. Can you tell me in your own words what a placebo is?
2. Why do you think that some research trials use a placebo?
3. Do you think it is possible to have side effects if you are taking a placebo? Why?
4. How did you feel when you heard that you/your child might be taking a placebo in this study?
5. If it turns out that you/your child have/has been taking the placebo, how will that make you feel?
6. If it turns out that you/your child have/has been taking the actual antibiotic (levofloxacin), how will that make you feel?

Activity:

Equipment:

- 2 plastic/paper cups (not transparent)
- Two small containers, each must fit under the cup. One should be labelled P (placebo) and one L (Levofloxacin). The one labelled L should be filled with flour (representing the antibiotic) and the other should be empty.

Steps:

1. Show participant the two small containers. Explain to them that one represents the placebo and represents the antibiotic, levofloxacin.
2. Turn the two cups upside down and place one container under each cup. Quickly switch the cups round multiple times until no one is sure which cup hides which container. Ask the participant to choose a cup but not to turn it over.
3. Explain to the participant that the study is like this. The study is double-blinded and placebo-controlled. No one knows what is under the cup. No one knows if [you are] taking levofloxacin or placebo.
4. Ask the participant: “If in the study, you could choose either the levofloxacin or the placebo for yourself/your child, which would you chose? Why?”
5. Tell the participant to turn over the cup. Ask the participant:
   1. Which one did you get?
   2. How do you feel about getting the placebo/levofloxacin?
   3. How would you have felt if you had gotten the other one?
6. Say to the participant: This is just a game. Would you like to know at the end of the study whether you/your child were actually taking the placebo or the antibiotic (levofloxacin)?
7. Ask the participant: Would you like to know the outcome of the study i.e., would you like to know whether levofloxacin actually works to prevent TB in children living with someone who has MDR-TB? What would be the best way for us to let you know?

End by explaining to the participant that we don’t really know if the study drug works to prevent TB and we do not know everything about levofloxacin (the active ingredient) and whether might cause side effects in some children. It is important that both the study staff, the caregivers and the children don’t know what the child is taking. If they did, it might influence how the staff treat the children, and it might influence how well the children take the study drug. It is important for the study that we can compare children who are taking the actual antibiotic, with children who are not.
