# Supplementary material for: Holistic acceptability of an adult levofloxacin formulation in children and adolescents on a tuberculosis preventive treatment trial
Source: PLOS Glob Public Health. 2024 Jul 5;4(7):e0003381. doi: 10.1371/journal.pgph.0003381 (PMC11226063; doi:10.1371/journal.pgph.0003381)
Supplement: Attachment — Submitted filename: Response to reviewers.docx [file pgph.0003381.s002.docx]

21 March 2024

Dear Editor

Thank you for the opportunity to revise this manuscript and to the reviewers for their constructive comments. Please see our comments to the reviewers (in bold) below.

Kind regards

Dr Susan Purchase

**Reviewer's Responses to Questions**

Comments to the Author

1. Does this manuscript meet PLOS Global Public Health’s publication criteria? Is the manuscript technically sound, and do the data support the conclusions? The manuscript must describe methodologically and ethically rigorous research with conclusions that are appropriately drawn based on the data presented.

Reviewer #1: Yes

Reviewer #2: Yes

2. Has the statistical analysis been performed appropriately and rigorously?

Reviewer #1: N/A

Reviewer #2: N/A

3. Have the authors made all data underlying the findings in their manuscript fully available (please refer to the Data Availability Statement at the start of the manuscript PDF file)?

The PLOS Data policy requires authors to make all data underlying the findings described in their manuscript fully available without restriction, with rare exception. The data should be provided as part of the manuscript or its supporting information, or deposited to a public repository. For example, in addition to summary statistics, the data points behind means, medians and variance measures should be available. If there are restrictions on publicly sharing data—e.g. participant privacy or use of data from a third party—those must be specified.

Reviewer #1: Yes

Reviewer #2: No

**Full interviews and case descriptions were not deposited into a public repository. Due to level of detail and quotes included, there is a possibility participants could be identified from the data. Our informed consent documents specified that only the research team would have access to the raw data.**

4. Is the manuscript presented in an intelligible fashion and written in standard English?

PLOS Global Public Health does not copyedit accepted manuscripts, so the language in submitted articles must be clear, correct, and unambiguous. Any typographical or grammatical errors should be corrected at revision, so please note any specific errors here.

Reviewer #1: Yes

Reviewer #2: Yes

5. Review Comments to the Author

Reviewer #1: This is an excellent paper on an important topic, and the authors have done exceptional work including a qualitative acceptability study within a larger trial of TPT. The longitudinal nature of the interviews is a great strength, and it is also exciting to see that they included interviews with both younger and older children.

There are some areas of the paper, however, that need to be strengthened.

First, the authors cite the study by Wademan et al as the conceptual framework for the data analysis. This is a strong framework to use, but some readers may not be familiar with it. It would help (if there is space) to include the figure from this paper to guide the reader. It would also greatly strengthen the paper to have the discussion section organized around this framework and to include a figure of the findings as they related to each of the domains of acceptability identified in Wademan's paper. The Wademan framework is really seminal in the pediatric TB space. I know the framework well and understand the authors are presenting many of the acceptability aspects in the results/discussion section. But for readers not as familiar with the framework, the results seems a bit like a "laundry list" of issues. It is always a struggle with word limits and qualitative data, and a figure (with some examples/sample quotes) showacse the findings in the different acceptability domains would be a strong addition to the paper.

**Thank you for this valuable observation. We have included a figure which incorporates both Wademan’s framework and our key interview results. We trust this will help readers who are not familiar with the framework and will also highlight key findings.**

Second, I am not sure why the authors seem to overly focus on the dug formulation in the paper. I know this is an important issue, but it seems to me the authors did key work on the acceptability of TPT as a whole. And I think this is important to highlight in addition to the formulation work. TPT is often viewed as a secondary priority for programs, but for families affected by TB and DR-TB, prevention seems like a key priority from the data you present. I would highlight this more and not just focus on the formulation.

**Thank you for this. We will highlight what we found regarding TB preventive therapy (TPT) generally in the new figure and in the discussion. However, although we set out to understand participants perceptions of TPT as a whole, most had very little direct experience of or knowledge of TPT as provided in routine care. Many felt they knew almost nothing about TPT, aside from what they had been told by study staff. The concept of TPT and infection versus disease is already complex, and study participants also needed to understand the concept of placebo in this trial. These issues made separation of acceptability of the study drug and acceptability of TPT generally quite difficult, especially in those who had no prior experience of TPT. We report findings based on our participants’ response to questions regarding the use of TPT and prevention more broadly, see lines 200-208.**

Third, while the focus of the paper (based on the title and framing) seems to be on the acceptability of the adult formulation, it is not clear to me why the authors feel it is necessary to see if adult formulations are acceptable to children. Data from multiple other diseases show that they clearly are not. So why do we need to prove this again in TB? I applaud the authors for doing this work and collecting the data, but I think they need to provide more framing and support for why we need data on the unacceptability of adult formulations for children. Some of this is about logistics and advocacy. But this should be acknowledged in the discussion--TB is way behind. There has been important work on pediatric formulation development in recent years in TB, however, and the authors should cite and acknowledge this more. The reader is also left to wonder why this study did not use the available pediatric formulations of levofloxacin (which were available for most of the study) or even try to compare the adult and the peds formulation. If this was for logistical reasons, this should be acknowledged more and discussed in the paper, as it seems like a hole in the study.

**Thank you for these important comments.** **The novel 100mg dispersible formulation was not used in the trial due to results from a lead-in pharmacokinetic trial (Garcia-Prats et al., 2019) indicating much lower bioavailability of the adult formulation compared with the dispersible formulation, and subsequent concerns regarding correct dosing in young children. Although previous work did indicate improved acceptability of the dispersible formulation (*Purchase et al., 2019 Acceptability of a Novel Levofloxacin Dispersible Tablet Formulation in Young Children Exposed to Multidrug-resistant Tuberculosis*), this work was done in a small group of very young children (median age 1.9 years). As the adult formulation of levofloxacin is considerably cheaper than the dispersible formulation, and well known to government procurement agencies and clinicians, it is used widely in children of all ages, and likely will still be used for many years to come. We felt it was important to collect extensive, longitudinal quantitative and qualitative acceptability on the adult formulation, as part of TB-CHAMP. Interestingly, initial data (unpublished) from the quantitative work shows relatively good acceptability of the adult formulation (87%** **good acceptability on a composite acceptability score by week 24), and even better acceptability (93% good acceptability on a composite acceptability score by week 24) in children able to swallow tablets whole. Children on TB-CHAMP were able to swallow the tablet whole/halved at a much younger age than anticipated (65% of 3–5-year-olds could swallow whole/halved). We feel the adult levofloxacin formulation still has a place in TB prevention programmes for children, especially in the slightly older age groups. Although not a finding in this study, we also feel choice is particularly empowering for participants and caregivers, and gives a sense of agency. For those programmes where there are major budget concerns, very young children and children who struggle to swallow the tablet whole could be offered a dispersible formulation, with adult formulation offered to those easily able to swallow. Please see lines 62-65 for updates to the text in this regard.**

Some smaller comments are also below:

1. The authors should add a reference on the topic of saturation. This topic is somewhat controversial in qualitative research, and it should be referenced in the paper.

**Thank you, this has been done. See lines 100-102.**

2. Is there a way to share the interview guides? I did not see them with the paper. Perhaps as an annex?

**Thank you – will submit as supplementary file.**

3. Lines 219-220 seem to be out of place here or missing some words?

**We have added some words which will hopefully clarify. Folders going missing occurs regularly in primary health clinics in South Africa. Clinicians will not see patients until the folder has been found or a new one made, and this often results in very long waiting times for patients. Interview participants highlighted this as a primary frustration when accessing preventive therapy in routine care. Please see lines 254-256.**

4. Lines 232-233 refer to a "lack of caregiver ambition...". The authors should rethink this sentence. The previous sentences describe aspects of depression in people (sleeping for many hours, anhedonia). To see it called a "lack of ambition" seems a bit of a mis-judgement that could inadvertently contribute to stigma.

**Thank you for pointing this out – the wording has been changed.**

Overall, this is a strong paper that will add a great deal to field should the authors be able to make these revisions.

Reviewer #2: This is an important and well written paper about a sub-study of the TB-Champ trial by a very experienced and knowledgeable team both on clinical/clinical research aspects and acceptability studies in the field of childhood MDR TB treatment. The TB-Champ trial, together with the V-QUIN trial recently provided evidence that levofloxacin can prevent development of MDR-TB in household contacts of adults with MDR-TB (results presented at Union conference). Access to MDR TB drugs in children for TPT and MDR Tx is a key issue.

I only have a minor comments to consider.

1. Interviews and results are touching on many aspects, with a broad vision of acceptability, not restricted to palatability/acceptability of levofloxacin tablets per se, in line with Wademan’s conceptual framework. I suggest taking that in consideration in the title, which for me reads as focusing very much on the adult levofloxacin formulation hence on the 1st part of the results.

**Thank you for this insight. As participants only had experience of the adult formulation, we did not want to remove this from the title, but have included the word “holistic” to try and highlight the broad nature of our enquiry.**

2. I suggest adding a short description of the instructions that were given to parents/children on how and when to take the pills as well as on the treatment education package received which may have influenced the perceptions that study participants had of their treatment. Results section includes a description on how caregivers administered the treatment but it would be helpful to understand what instructions were given to them.

**Thank you. This has been added to the methods section under Treatment (see lines 90-96). Parents/children were not given any specific treatment education package, but did use a treatment adherence card.**

3. Could authors clarify how they did purposive sampling/checked that they reached saturation with the case study longitudinal design? Was the 1st visit considered or did they follow children till the third interview to decide whether other recruitments were done (which would seem more challenging from a practical point of view).

**We have added the following details to the ‘sampling strategy’ section (see lines 99-108):**

**We used maximum variation purposive sampling to maximize diversity in experience, and to ensure a representative sample of females and males, and age and language groups. We sampled to a priori thematic saturation (the point at which no new information relating to the conceptual framework themes covered was being obtained from data collection, or new participants) (Saunders et al., 2017). This point was determined by the socio-behavioural researchers conducting the interviews in consultation with the senior researchers who were concurrently reading their case descriptions, and was based on the first interview, primarily for logistic reasons.**

4. Could authors clarify whether caregivers were the index case? This may also have influenced their perception of how the child dealt with treatment, although it appears from the interviews that within community stigma seemed limited.

**None of the caregivers interviewed were the index cases (although in the main study some caregivers were index cases). We have clarified this at the beginning of the Results section (see line 150).**

5. Fear of stigma is mentioned once but there does not seem to be major stigma related to MDR TB as compared to DS TB, at least not in the way it is reported here. In the authors’ opinion, could this be due to the trial context and to a “selected” study population or is the community cluster randomized design representative of what is happening in the community?

**Stigma did not seem a major problem, but caregivers did stress to friends and families that their children did not have TB, but were on preventive medication. Caregivers, however, used pejorative language when speaking of index cases. We also noted the existence of intersectional stigma (see lines 210-219). It was clear that index cases often did not disclose their status beyond the immediate household, and MDR-TB was certainly seen as a more dangerous disease. We have added wording to this effect (see lines 241-243). For children to be included in TB-CHAMP, the index case would have had to disclose at least to their household. This may have excluded index cases with high levels of internalised and anticipated stigma.**

6. At last, in the discussion, authors refer to the better acceptability of levofloxacine 100 mg. Was this considered as an alternative in the trial? If no why not?

**This was certainly considered. The novel 100mg dispersible formulation was not used in the trial due to results from a lead-in pharmacokinetic trial (Garcia-Prats et al., 2019) indicating much lower bioavailability of the adult formulation compared with the dispersible formulation, and subsequent concerns regarding correct dosing of the dispersible formulation in young children. A recent randomised, open-label cross-over trial of the relative bioavailability of levofloxacin dispersible vs crushed non-dispersible tablets confirms these findings and recommends review of current dosing in children for both the dispersible and non-dispersible tablets (van der Laan et al., 2023). Acceptability work done as part of this trial and recently published (Wademan et al., 2024) indicates that caregivers and young children preferred the dispersible formulation. It is important to note, however, that it was compared to the crushed adult formulation. Results from quantitative acceptability work done in TB-CHAMP shows that children who can swallow the levofloxacin tablet whole find it very acceptable. As 65% of children aged 3-5 years were able to swallow the tablet whole, this cheap and easily available formulation should not be overlooked in preventive therapy programs for children.**

6. PLOS authors have the option to publish the peer review history of their article (what does this mean?). If published, this will include your full peer review and any attached files.

Do you want your identity to be public for this peer review? If you choose “no”, your identity will remain anonymous but your review may still be made public.

For information about this choice, including consent withdrawal, please see our Privacy Policy.

Reviewer #1: No

Reviewer #2: Yes: Olivier Marcy

[NOTE: If reviewer comments were submitted as an attachment file, they will be attached to this email and accessible via the submission site. Please log into your account, locate the manuscript record, and check for the action link "View Attachments". If this link does not appear, there are no attachment files.]

While revising your submission, please upload your figure files to the Preflight Analysis and Conversion Engine (PACE) digital diagnostic tool, https://pacev2.apexcovantage.com/. PACE helps ensure that figures meet PLOS requirements. To use PACE, you must first register as a user. Registration is free. Then, login and navigate to the UPLOAD tab, where you will find detailed instructions on how to use the tool. If you encounter any issues or have any questions when using PACE, please email PLOS at figures@plos.org. Please note that Supporting Information files do not need this step.

**Done**
